# Social justice in Australian and Canadian dietetic regulatory documents: a content analysis

**DOI:** 10.1017/S1368980026102493

**Published:** 2026-04-10

**Authors:** Olivia Gray, Ellen Wynn, Jennifer Brady, Annabelle Wilson

**Affiliations:** 1 College of Nursing and Health Sciences, Flinders University, Adelaide, Australia; 2 College of Medicine and Public Health, Flinders Health and Medical Research Institute, Flinders University, Adelaide, Australia; 3 School of Nutrition and Dietetics, Women’s and Gender Studies, Acadia University, Wolfville, Canada

**Keywords:** Social justice, Dietetics, Policy, Public health nutrition, Content analysis

## Abstract

**Objective:**

This research was conducted to determine if and how Australian and Canadian dietetic regulatory bodies incorporate social justice into regulatory documents and how this compares between two otherwise demographically and politically similar countries.

**Design:**

Quantitative and qualitative content analysis of Australian and Canadian dietetic regulatory documents was performed to determine how often and in what context social justice terms were incorporated into dietetics regulation.

**Setting:**

Australia and Canada

**Participants:**

Regulatory documents in Australia and Canada

**Results:**

Findings reveal that social justice is framed differently between the two countries, particularly related to working with people who experience marginalisation. Regulatory documents seldom addressed issues of systemic injustice, focusing instead on self-awareness and individualistic approaches to care.

**Conclusions:**

Social justice is currently framed in nutrition and dietetics regulatory documents in ways that do not align with core principles of social justice. Social justice should be reframed in regulatory documents to shift attention away from awareness, towards action, and should be done in a way that addresses systemic injustices in healthcare. Developing a clear and consistent definition of what social justice is is a critical first step in achieving this goal to overcome the challenges identified in this research study.

Social justice is highly contextual and multifaceted^(1,2)^. While there is no single definition for what social justice entails, most descriptions include fairness for all people, redistribution of resources to improve situations of those experiencing disadvantage and critically examining systemic structures giving rise to disparities amongst different social groups^(1,3,4)^. Social justice is vital for public health nutrition because it supports improving population health by addressing inequalities, ensuring equitable access to healthy food and environments for all people and reorienting health services to address health inequities^(3,5)^. However, a combination of inadequate social policies, programmes and politics further affects people experiencing marginalisation who are already disproportionately impacted by health disparities and social injustices^(6–8)^. Furthermore, health professionals trained in Western education institutions are traditionally taught to practise in ways that privilege a positivist, biomedical approach to health which has limited acknowledgement of systemic factors that influence health, such as the social determinants of health, which are central to advancing public health nutrition^(9)^. Dietitians are key health professionals who contribute to public health nutrition through their involvement in the development and implementation of public health interventions. They are the only regulated nutrition professionals approved to work clinically with individuals. However, dietitians also work in a range of community and public health settings, alongside other non-dietetic professionals such as nutritionists. This means that regulation of the dietetics profession is highly important to achieving appropriate public health nutrition outcomes across the spectrum of areas in which nutrition impacts health and wellbeing of the population. As such, there is a need to ensure that dietitians have the knowledge, skills and capacity to practise in socially just ways so that they can contribute to advancing social justice in public health nutrition. Dietitians in Australia and Canada are regulated by profession-specific regulatory bodies who produce regulatory documents that outline the expected knowledge, scope and practice of dietitians. Examples of these documents include Code of Ethics and Code of Conduct. Such documents are used to guide professional education, regulation and licensure. In this paper, the term ‘regulatory document’ refers to any document produced by a regulatory body that states the expected practice, knowledge and/or scope of dietitians.

An analysis of dietetics policy documents in the USA found that dietetics regulatory documents lacked acknowledgement of several social justice issues, particularly in relation to the gendered history of dietetics and the need to recognise gender dynamics that uphold power hierarchies in the workplaces^(10)^. Furthermore, Canadian researchers have called for adaptations to dietetics education and training, to support the development of emerging dietitians who feel confident to advance social justice through their practice^(11)^. Limited research on social justice in other professions related to public health, such as social work and nursing, has been undertaken^(12,13)^. However, given that dietitians are qualified nutrition professionals working in public health nutrition as well as implementing public health actions, it is imperative that social justice in this profession is understood^(14)^. Currently, no research exists that explores how social justice is incorporated into Canadian and Australian dietetics regulatory documents. This represents a challenge in the regulation and training of emerging dietitians, as regulatory documents guide training competencies and assessment and ultimately determine if dietitians are accredited by their governing body. As such, this study sought to determine if and how social justice is currently incorporated into dietetics regulatory documents in Australia and Canada. These countries were chosen based on sociodemographic similarities and because of similarities in dietetic professional regulation and areas of practice (e.g. clinical, community, private practice, public health and health promotion). The findings from this research contribute to building capacity of the public health nutrition workforce, through investigating the systemic structures that determine if and how social justice is incorporated into dietetic regulation. While specific to dietitians, this novel research paves the way forward for researchers and public health advocates to advance social justice by ensuring practitioners working in public health nutrition are equipped to advance social justice through their professional training, education and regulation.

## Methodology and methods

### Epistemology, theoretical perspective, methodology and method

This research was conducted in alignment with constructionist epistemology, in which the authors believe that meaning is constructed and interpreted through social interactions and influences^(15)^. A critical theory approach was applied to elucidate how power inequities manifest and operate through the content included in dietetics regulatory documents^(16)^. By taking a critical theory approach, authors aimed to challenge oppression and promote social justice through seeking to uncover what inequities may inadvertently exist within dietetic regulatory documents. Issues of social justice have been said to motivate critical theory, thus highlighting the alignment of this approach with the research aims and purpose^(17)^. Furthermore, this is important because a critical theory perspective allowed identification of what concepts associated with social justice are and are not identified in key documents guiding the dietetics profession. This has implications for what students and dietitians are exposed to in their learning and practice. Document analysis was the methodology used for this research^(18)^. Conventional qualitative content analysis was the method used to undertake analysis^(16,18)^. A small number of quantitative approaches were also used (frequency counts). This is described in more detail in the following sections.

### Identification of documents

Purposeful sampling was used to identify Australian and Canadian regulatory documents which provide guidance for how dietitians should practise and standards for practice, as well as for dietetic education (e.g. accreditation documents). This data sampling method aligns with policy analysis data processes used in similar studies^(10,19)^. All documents were publicly available and accessed through national or provincial regulatory body websites. Australian documents were primarily identified through ‘Dietitians Australia’, and Canadian documents were identified through searching ‘College of Dietitians of (province of interest)’. Bodies with regulatory influence, for example, the Dietitian and Nutritionist Regulatory Council (Australia) and the Partnership for Dietetic Practice (Canada) were also searched for relevant documents. The websites of each organisation relevant to dietetic regulation were hand-searched through navigating pages providing access to ‘resources’, ‘publications’, ‘corporate documents’, or similar. All documents identified were compiled and presented to the research team. Identification of suitable documents was determined through conversations between two of the authors who have extensive experience with regulation of the dietetics profession in each country. The research team reviewed and discussed documents until a consensus was reached on which documents should be included, based on their relevance to the research aims. Only documents written in English and the most current version of each regulatory document were included in the content analysis as the researchers sought to understand how dietetics regulatory bodies currently approached social justice issues. All included documents were analysed using Microsoft Excel and NVivo Version 13 software through both quantitative and qualitative content analysis techniques^(20,21)^. The documents from each country were analysed separately to provide insight into how each country framed social justice. This allowed findings to be compared across both countries.

### Quantitative content analysis

Quantitative content analysis was performed first and examined the frequency of social justice terms in regulatory documents to indicate the principles of social justice included. In preparation for conducting the frequency counts, two research team members developed a list of social justice-related terms and phrases. This list of terms was developed from a critical theory stance, whereby authors sought to challenge any potential social inequities that may be present. An existing list of terms from similar research was adopted as a starting point, and then the extant, albeit limited, literature investigating social justice issues in dietetics was scoped for additional terms^(22)^. Researchers also suggested and discussed terms based on their personal experiences as dietitians working in public health-related areas. The list was also inductively developed as analysis occurred, given that there is limited published literature on how to identify social justice terminology or undertake a study of this nature. New social justice terms were added as they appeared in the documents already identified as eligible for inclusion. After the addition of new terms to the list, previously examined documents were reanalysed for newly added terms. The master list of terms was adapted to each country’s context. For example, ‘Aboriginal and Torres Strait Islander’ was used only in the Australian frequency counts. Similarly, ‘BIPOC’ (a term commonly used in North America to mean Black Indigenous Person of Color) was included only for the Canadian counts. The differences in terms used for each country were determined through discussion between the research team, which included both Canadian and Australian dietitian-researchers who are experienced in social justice research and dietetics advocacy. Terms were also adjusted for spelling differences used between countries, for example, interchanging ‘s’ (for Australia) and ‘z’ (for Canada) in words such as ‘colonization’. Table 1 displays the master list of the eighty-one social justice terms and phrases used in the frequency counts.

**Table 1.** Master list of social justice search terms used to conduct frequency counts across Australian and Canadian dietetic regulatory documents

Ability	Culturally appropriate	Gender roles	Power
Able-bodied	Culturally competent	Gender socialisation	Prejudice
Ableism	Culturally humility	Health equity	Privilege
Aboriginal and Torres Strait Islander	Culturally responsive	Homophobia	Race
Advocate/advocacy	Culturally safe	Gender identity	Racialised
Age	Disability	Inclusion/inclusive	Racism
Ageism	Discrimination	Indigenous	Religion
Ally	Disparity	Inequality	Sexual orientation
Ancestry	Diverse/diversity	Inequity	Social construction
Bias/biases	Equality	Intersectionality	Social determinants of health
BIPOC (Black Indigenous Person of Colour)	Equity	Intersex	Social gradient
Cisgender	Ethnic background	Justice	Social justice
Classism	Ethnicity	LGBT	Social stratification
Client-centred	Ethno-cultural	Nationality	Spirituality
Colonisation	First nations	Non-judgemental	Stereotypes
Critical	Food insecurity	Nutrition security	Structural competence
Critical thinking	Food justice	Oppression	Sustainability
Cultural competence	Food security	Patient-centred	Transphobia
Cultural identity	Food sovereignty	Person-centred	Two-spirit
Cultural safety	Gender	Queer	Wealth

Frequency counts were conducted using the ‘text search’ function in Nvivo^(21)^. All included documents were individually searched for each social justice term included in the master list (Table 1). If and when a new term was added to the term list, all documents were then researched for that term. The primary author reviewed each match to ensure it was used in a way that was relevant to social justice, thus contributing to a deeper understanding of how social justice is incorporated into dietetics regulatory documents^(23)^. Text search matches were only included in frequency counts if they (1) were framed in a way that was relevant to social justice principles and (2) were discussed in the context of dietetics practice. This step was crucial to ensuring the research was credible and reliable, as several terms included in the master list of search terms (Table 1) can be used in varying ways. For example, ‘advocate’ can be used in the context of advancing the profession (e.g. ‘advocates for dietetics practice, and clinical standards’) and in the context of social justice-related issues (e.g. ‘advocates for clients from diverse backgrounds, and their unique healthcare needs’). This process also allowed the authors to exclude any terms that appeared in reference lists, which may otherwise have skewed the results. All appropriate matches were collated in a Microsoft Excel spreadsheet. Two researchers (one from Australia and one from Canada) conducted frequency counts for their respective country’s regulatory documents. The findings were then shared with the broader research team and compared by the primary author.

### Qualitative content analysis

Qualitative content analysis was then conducted using Hsieh and Shannon’s (2005) conventional content analysis to provide insight into how social justice was included and framed in dietetic regulatory documents^(18)^. This allowed us to derive coding categories directly from the text. This approach is useful when there is a lack of existing theory or research about a particular topic, which is the case in this research^(18)^. The steps taken for conventional qualitative content analysis followed those outlined by Hsieh and Shannon (2005)^(18)^. The analysis was first conducted by the primary author. This included reading the data to become immersed in it and understand it as a whole. Codes were then derived by reading data word for word and highlighting exact words which demonstrated key concepts. Notes about initial thoughts were then made, and labels were given to codes which consisted of more than one thought. After initial coding, the primary author presented the labels to the research team for reflection and revision. Links between codes were then identified, and codes were sorted into themes based on these links. Preliminary themes were presented to the research team for discussion and review before the primary author refined and named the final themes reported.

### Reflexivity and trustworthiness of data

Trustworthiness of data was managed through researcher reflexivity. This was incorporated into the research project through discussion about codes and themes with the research team. Using quantitative and qualitative content analyses supported the credibility of the findings reported because the results from the qualitative content analysis provided context around the use of the term searched for in the quantitative content analysis and confirmed that it was related to social justice. This fits with the process described by Hsieh and Shannon (2005) where content analysis examines the extent to which words are used in a text and then analysis occurs about how the words are used. Ethics approval was not required as all data were obtained from published documents that are accessible to the public online.

## Findings

A total of thirty-four documents, thirteen from Australia and twenty-one from Canada, were identified for potential inclusion in this analysis. Eight Australian documents were excluded as they did not meet the inclusion criteria. Two Canadian documents from Quebec were excluded because they were unavailable in the English language. As a result, twenty-four regulatory documents were included in this research study (Table 2).

**Table 2.** Documents included in content analysis from Australia and Canada

Country	Document name and date of publication	Publishing body
Australia	National Competency Standards for Dietitians (2021)	Dietitians Australia
	Code of Conduct for dietitians and nutritionists (2023)	Dietitians Australia
	Scope of practice (n.d.)	Dietitians Australia
	Accreditation Standards for Dietetics Education Programs (2022)	Australian Dietetics Council
	Self-Regulating Health Profession Peak Bodies Membership Standards (2016)	National Alliance of Self-Regulating Health Professions
Canada	Code of Ethics (2008)	College of Dietitians of Alberta
	Standards of Practice (2018)	
	Code of Ethics (2006, revised 2012)	College of Dietitians of British Columbia
	Standards of Practice (2019)	
	Code of Ethics for Registered Dietitians (2005)	College of Dietitians of Manitoba
	Standards of Practice (2023)	
	Code of Ethics (2007)	New Brunswick Association of Dietitians
	Standards of Practice (2007, revised 2015)	
	Code of Ethics (2019)	Newfoundland and Labrador College of Dietitians
	Standards of Practice (2016)	
	Code of Ethics (2007, amended 2020)	Nova Scotia Dietetic Association
	Standards of Practice (2020)	
	Code of Ethics (2005)	Saskatchewan Dietitians Association
	Standards of Practice (2018)	
	Code of Ethics	College of Dietitians of Ontario
	Standard for Dietitians Practicing through Delegation of Controlled Acts (2020)	
	Standards and Guidelines for Professional Practice Conflict of Interest (2017)	
	Standard Consent to Treatment and for the Collection, Use and Disclosure of Personal Health Information (2019)	
	Professional Practice Standards for Record Keeping (2017)	
	Collecting Capillary Blood Samples through Skin Pricking & Monitoring the Blood Readings (n.d.)	
	Virtual Care Standards and Guidelines for Dietitians in Ontario (n.d.)	
	Social Media Standards and Guidelines (n.d.)	
	Code of Ethics (2019, Revised 2021)	College of Dietitians of Prince Edward Island
	Standards of Practice (2019, Revised 2021)	
	Integrated Competencies for Dietetic Education and Practice Version 3.0 (2020)	Partnership for Dietetic Education and Practice

### Quantitative content analysis

Table 3 presents findings from the quantitative content analysis and displays the frequency of social justice terms included across all regulatory documents. The master list of terms (Table 1) contained eighty-one terms. Thirty-eight of these terms were included in the sample of Australian documents (*n* 5), and forty-three were included in the sample of Canadian documents (*n* 19). Most terms were incorporated less than ten times across the sample of Australian and Canadian regulatory documents. The most frequently (> 10 times) included terms in Australian documents were related to culture and race and included ‘cultural safety’ and ‘Aboriginal and Torres Strait Islander’ among others. The most frequently (> 10 times) included terms across the Canadian documents were related to race, culture, religion and diversity and included ‘nationality’, ‘Indigenous’, ‘diversity’, ‘sexual orientation’, ‘power bias’, ‘client-centred’ and ‘religion’.

**Table 3.** Total frequency of social justice terms included in Australian and Canadian dietetics regulatory documents

Australian documents				Canadian documents			
Social justice term	Frequency	Social justice term	Frequency	Social justice term	Frequency	Social justice term	Frequency
Age	1	Power	2	Advocacy	1	Cultural humility	5
Critical	1	Race	2	Colonisation	1	Cultural safety	5
Cultural competence	1	Religion	2	Cultural competence	1	Discrimination/discriminatory	5
Equity	1	Social determinants of health	2	Culturally competent	1	Racism	6
Ethnicity	1	Sustainability	2	Disparity/disparities	1	Social justice	6
First nations	1	Culturally appropriate	2	Equality	1	Ability	7
Food security	1	Colonisation	3	Gender identity	1	Determinants of health	7
Gender identity	1	Discrimination	3	LGBT	1	Inclusion	7
Health equity	1	Disparity	3	Privilege	1	Equity/equitable	8
Justice	1	Inequality	3	Racialised	1	Advocate	9
Non-judgemental	1	Nutrition security	3	Social determinants	1	Ancestry	9
Person-centred	1	Gender	3	Wealth	1	Disability	9
Prejudice	1	Bias(es)	4	Critical thinking	2	Ethnic background	9
Privilege	1	Racism	5	Food insecurity	2	Nationality	10
Social justice	1	Advocate/advocacy	6	Gender	2	Indigenous	12
Culturally responsive	1	Client-centred	6	Social class	2	Diversity/diverse	14
Cultural safety	2	Diverse/diversity	8	Transphobia	2	Power	15
Disability	2	Culturally safe	12	Food security	3	Bias	17
Inclusion/inclusive	2	Aboriginal and Torres Strait Islander	26	Culturally safe	4	Sexual orientation	18
				Ethnicity	4	Client-centred	21
				Ethno-cultural	4	Religion	24
				Race	4		

The total frequency counts included term repetition in a document. For example, the term ‘culturally safe’ was identified twelve times in total across the five Australian documents, indicating repetition of the term both across and within documents. Table 4 outlines the total number of social justice terms per dietetic regulatory document included. Social justice terms featured between three and seventy-five times in Australian documents, with the Code of Conduct for dietitians and nutritionists containing the most (*n* 75) social justice terms. Social justice terms were incorporated between two and sixty times across the Canadian documents, with the Ontario Standards for Dietitians Practicing through Delegation of Controlled Acts containing the most (*n* 60) social justice terms, followed closely by the Integrated Competencies for Dietetics Education and Practice (*n* 58).

**Table 4.** Total number of social justice terms included in Australian and Canadian dietetics regulatory documents

Dietetic regulatory documents		
Country	Document name	Total number of social justice terms in document
Australia	Accreditation Standards for Dietetics Education Programs	5
	Code of Conduct for dietitians and nutritionists	75
	National Competency Standards for Dietitians	34
	Scope of practice	3
	Self-Regulating Health Profession Peak Bodies Membership Standards	3
Canada	Alberta Code of Ethics	9
	Alberta Standards of Practice	9
	British Columbia Code of Ethics	8
	British Columbia Standards of Practice	8
	Manitoba Code of Ethics for Registered Dietitians	12
	Manitoba Standards of Practice	2
	New Brunswick Code of Ethics	7
	New Brunswick Standards of Practice	2
	Newfoundland Code of Ethics	11
	Newfoundland Standards of Practice	7
	Nova Scotia Code of Ethics	11
	Nova Scotia Standards of Practice	9
	Ontario Code of Ethics	10
	Ontario Standards (All Seven Merged) for Dietitians Practicing through Delegation of Controlled Acts	60
	Prince Edward Island Code of Ethics	11
	Prince Edward Island Standards of Practice	8
	Saskatchewan Code of Ethics	12
	Saskatchewan Standards of Practice	8
	Integrated Competencies for Dietetic Education and Practice Version 3.0	58

### Conventional qualitative content analysis

**Figure 1.**
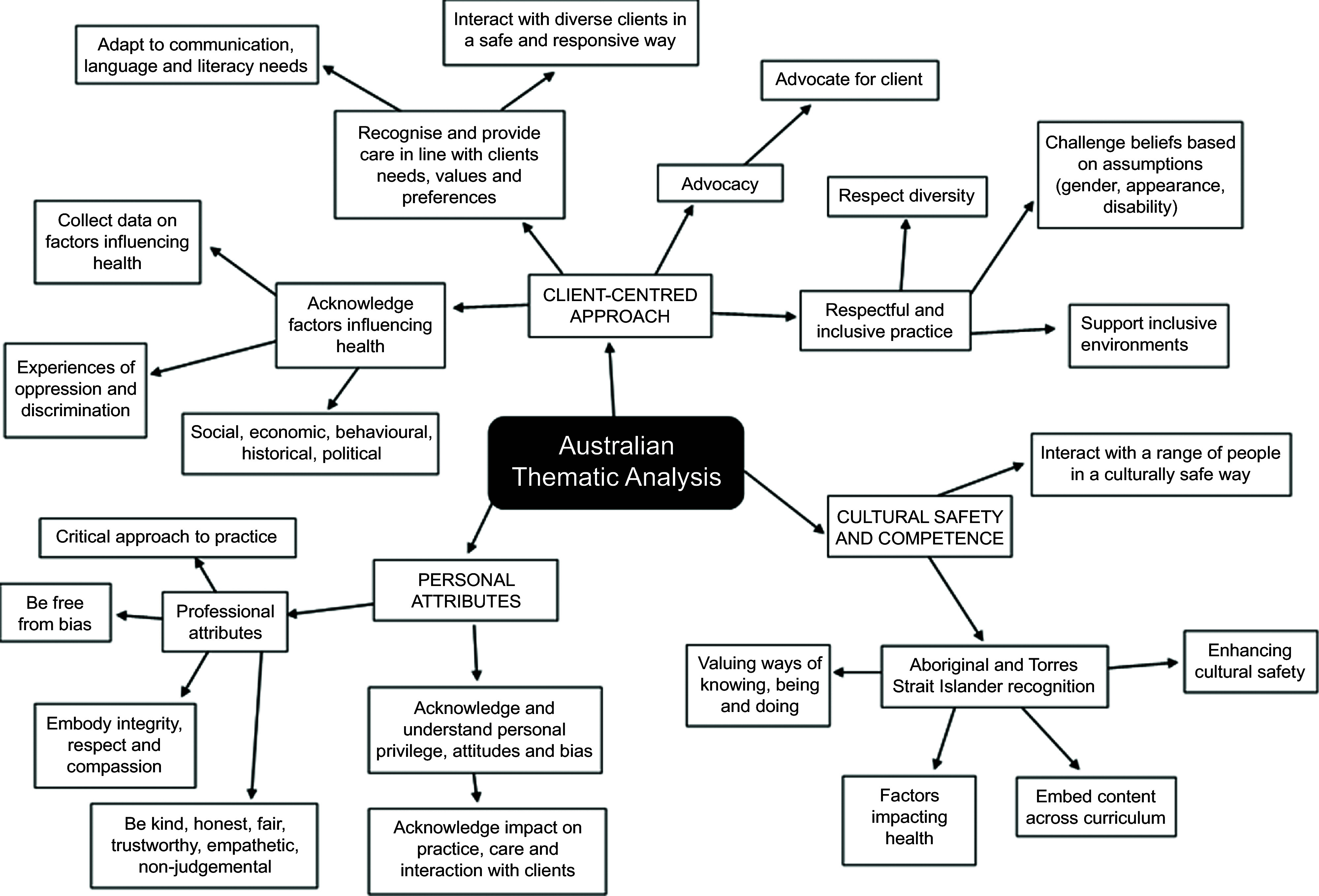
Mind map of themes generated from content analysis for Australian dietetic regulatory documents.

**Figure 2.**
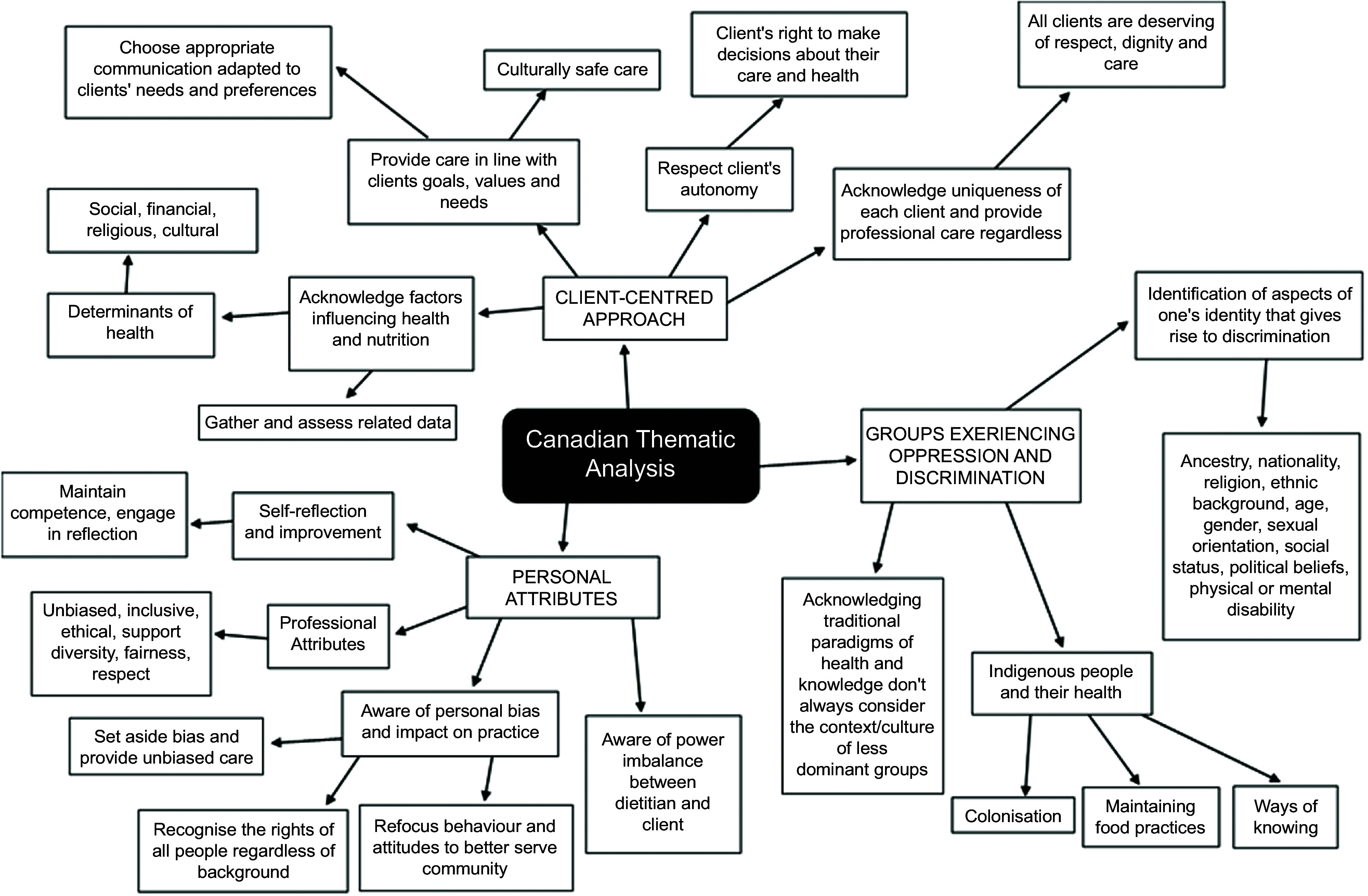
Mind map of themes generated from content analysis for Canadian dietetic regulatory documents.

Three themes were developed from the conventional qualitative content analysis for each country. Two of these themes occurred in both Australian and Canadian regulatory documents: ‘personal attributes’ and ‘client-centered approach’. The remaining themes were specific to each country. For Australia, the third theme was ‘cultural safety and competence’, and for Canada, the third theme was ‘groups experiencing oppression and discrimination’. Figures 1 and 2 show how these themes were developed and the breadth of concepts encapsulated within them.

### Personal attributes

Expected personal attributes of dietitians were often incorporated into regulatory documents for both countries. According to the documents analysed, dietitians in Australia and Canada should acknowledge personal bias and provide services in an unbiased manner. In particular, dietitians were expected to be self-aware of their personal biases, attitudes, values and privileges and how these impact their practice. For example, the Australian National Competency Standards (2021, p3) state that a competent dietitian: *‘Acknowledges, reflects on and understands own culture, values, beliefs, attitudes, biases, assumptions, privilege and power at the individual and systems level, and their influence on practice’*. Both Australian and Canadian documents went beyond this to address the role of power in client–dietitian relationships. However, this was more frequently recognised in Canadian documents as this content was often incorporated into the codes of ethics for each Province, with a statement similar, or identical, to: *‘The dietitian is sensitive to their position of relative power or influence in professional relationships…’* (Code of Ethics Alberta, 2008, p. 8). However, in Australian documents, only the Code of Conduct states: *‘recognise the inherent power imbalance that exists between dietitians and their clients and establish and maintain professional boundaries’* (Australian Code of Conduct, p. 17).

### Client-centred approach

Australian and Canadian dietitians were expected to approach their practice through a client-centred model of care. Regulatory documents prescribed that dietitians must provide respectful care to all people regardless of their unique backgrounds or attributes, which is a core pillar of dietetics practice in each of these countries. The qualitative content analysis showed that client-centred care should align with the client’s needs, values and preferences, and that dietitians should consider factors influencing the health and nutrition of their clients. For example: *‘A Dietitian provides quality professional services that reflect the unique needs, goals, values and circumstances of the client’* (Code of Ethics Alberta, 2007, p. 7; Code of Ethics Prince Edward Island, 2021, p. 6).

### Cultural safety and competence

Australian regulatory documents emphasised dietitians’ interactions with clients of diverse cultural backgrounds. Dietitians in Australia were expected to understand cultural needs and act in culturally safe and respectful ways, particularly towards Aboriginal and Torres Strait Islander Peoples. For example, the Australian dietitian’s Code of Conduct (2023, p13) states: *‘Provide dietetic services that align with Aboriginal and/or Torres Strait Islander peoples’ definition of health, free from bias and racism, which challenge beliefs based on assumptions, and are culturally safe and respectful for Aboriginal and/or Torres Strait Islander peoples’*. Several Australian documents also acknowledge diversity and discrimination, in relation to cultural safety: *“adopt practices that respect diversity, avoid bias, discrimination and racism, and challenge beliefs based on assumptions (for example, based on gender, disability, race, ethnicity, religion, sexuality, appearance, age or political beliefs),* Code of Conduct, p. 14.

### Groups experiencing oppression and discrimination

Many Canadian documents acknowledged specific aspects of clients’ identity and backgrounds that may give rise to discrimination. Dietitians were expected to provide services that catered to diverse groups of people regardless of their background. For example, several provincial codes of ethics stated: *‘The dietitian provides professional services in response to the client’s needs regardless of ancestry, nationality, ethnic background, religion, age, gender, social and marital status, sexual orientation, political beliefs, or physical or mental disability’* (Code of Ethics Alberta, 2008, p. 7; Code of Ethics Manitoba, 2005, p. 5; Code of Ethics Code of Ethics Newfoundland, 2019, p. 5; Prince Edward Island, 2021, p. 6; Code of Ethics Saskatchewan, 2005, p. 4). Similarly to Australian documents, this theme also encapsulated the need for dietitians to acknowledge Indigenous peoples’ ways of knowing and doing: *‘Demonstrate awareness of Indigenous values and ways of knowing related to health and wellness’* (Canadian Integrated Competencies for Dietetic Education and Practice Version 3.0, 2020, p. 14). However, the inclusion of content relating to Indigenous peoples in Canada was featured in only two documents: the Canadian Integrated Competencies for Dietetic Education and Practice Version 3.0 and the Ontario Standards of Practice.

## Discussion and international comparison

Previous research has not investigated the incorporation of social justice in dietetics regulation in Australia or Canada. This is a pertinent issue to public health nutrition as social justice is a core principle underpinning many issues addressed by public health nutrition professionals. The results reveal that in both countries, dietitians are expected to be self-aware of personal bias and attitudes and provide client-centred care, regardless of clients’ backgrounds. Key differences between Australian and Canadian regulatory documents related to the identification and inclusion of diversity-related issues. In Australia, there was a strong focus on Aboriginal and Torres Strait Islander inclusivity. There were two main places in the Australian documents where some other groups of people experiencing discrimination were mentioned. This includes the Code of Conduct and the Guide that accompanies the National Competency Standards (which was not included in this content analysis). In contrast, in documents from Canada, the acknowledgement of discriminatory factors, such as ethnicity, gender and disability, was broader and more overt across a wider range of documents. Overall, there was limited incorporation of social justice terms in the documents, with most individual social justice terms being incorporated less than ten times across the entire sample of Australian and Canadian regulatory documents, and most documents mentioning social justice terms less than twenty times per document. However, four documents contained a greater number of social justice terms compared to the rest of the sample, including the Code of Conduct for dietitians and nutritionists (Australia, *n* 75 terms), the Ontario Standards for Dietitians Practicing through Delegation of Controlled Acts (Canada, *n* 60 terms), the Integrated Competencies for Dietetics Education and Practice (Canada, *n* 58 terms) and the National Competency Standards (Australia, *n* 34). This suggests that there is inconsistent incorporation of social justice issues in dietetics regulatory documents, which may provide challenges for regulators and educators attempting to assess and measure dietitians’ competency for social justice action. This is particularly the case in Canada, where universal regulatory documents do not exist. This may be attributed to a lack of consistent definition of social justice that is contextualised to the dietetics profession, which has been identified as an issue in other research studies^(2,24)^. It is also important to acknowledge that the two regulatory documents that are most likely to guide dietetics education and regulation in Australia (the Code of Conduct and the National Competency Standards) did have the largest number of social justice terms, indicating that it may be more natural to place terms related to social justice in some documents more than others. However, despite this, we argue that for true incorporation of social justice across the profession and integration into education, it is vital to have such concepts embedded in all regulatory documents, even where it might not immediately seem obvious.

### Self-awareness of personal biases

Results from both Canadian and Australian content analyses showed consistent directives for dietitians to be self-aware and acknowledge their personal biases and attitudes. However, directives did not extend to dietitians taking action against injustice and, rather, suggested that dietitians ‘set aside’ these biases. Remaining in apolitical, unbiased stances ultimately perpetuates systems of inequity because no action is taken against injustice^(24,25)^. Furthermore, the concept of ‘setting aside’ one’s bias is controversial as social and cultural biases are absorbed and reflected through social structures and interactions, thus preventing one from being truly ‘unbiased’^(25)^. While self-awareness is an important starting point for enacting social change, being self-aware does not necessarily translate into action, as noted in several existing research studies^(12,24,26,27)^. As such, dietitians and public health nutrition practitioners must move towards social justice action through incorporating reflexive practices, critical learning opportunities, engaging in advocacy, and ultimately, transforming into socially just practitioners^(13,24,28)^. To do this, regulatory documents need to provide explicit directives for how dietetic qualified public health nutrition practitioners, and dietitians more broadly, can act in socially just ways and how they might work to identify and unravel these biases and how they impact on their work, rather than simply ‘putting bias aside’. Competencies identified as important for advancing social justice in the social work profession may provide a useful starting point for developing social justice competencies and guidelines for the public health nutrition discipline^(12)^. These include acknowledging oppression, identifying client strengths, empowering clients to be self-advocates when able and providing information, education or collaboration for support services in the community^(24)^. Furthermore, reframing competencies around social justice action, considering the factors identified by Hagar, Thomas & Reisch (2024) that contribute to social justice action in the broader population, may be useful for steering focus away from awareness towards action.

### Client-centred approach

Client-centred care is the dominant approach to care promoted in Australian and Canadian dietetic regulatory documents and was a prominent social justice theme for both countries (Figures 1 and 2). Client-centred care attempts to align healthcare professionals’ practices with the individual expectations and needs of clients^(29)^. This approach is supportive of social justice, through inherently incorporating non-discriminatory and inclusive approaches to practising^(27,29)^. However, the client-centred approach has also been critiqued in recent years, as it overlooks the role of external and systemic constraints that influence and ultimately determine health outcomes^(27,30)^. Individualistic care is dominant in neoliberal Western political systems such as Australia and Canada^(27,31)^. Continuing to promote individualistic care through concepts of ‘client-centred care’ may perpetuate systems of injustice if the concept is not expanded to address inequities perpetuated by social and systemic injustices^(27)^. As such, social justice competency in the context of client-centred care should be reframed to address the broader social structures that influence health, including families, communities, systems and institutions, rather than only considering individual action, which fails to address the broad spectrum of social justice issues in contemporary society^(30)^. We acknowledge that some documents, for example, the DA Code of Conduct, refer to the involvement of families and communities in client-centred practice. Additional information around how social justice can be addressed through working with families, communities and wider structures is an important way to expand upon this in future work.

### Identification of groups experiencing marginalisation

Australian and Canadian regulatory documents varied considerably in how they identified and discussed working with people experiencing marginalisation. Canadian documents broadly recognised groups of people experiencing discrimination or oppression based on factors of their identity, such as race, religion and gender. Conversely, Australian documents strongly focused on Aboriginal and Torres Strait Islander status as the main form of diversity among dietetics clients, with cultural safety as a strategy to address this. As such, ‘Aboriginal and Torres Strait Islander’ was the most frequently incorporated social justice term in Australian dietetic documents, while ‘religion’ and ‘sexual orientation’ were among the most frequently used social justice terms in Canadian documents. This may reflect the way in which the most recent Australian National Competency Standards were developed which involved a document analysis which resulted in four key areas being added, one of which was Aboriginal and Torres Strait Islander health^(32)^. In addition to retaining this focus on Aboriginal and Torres Strait Islander health, additional recognition of other groups experiencing injustice in more Australian regulatory documents may assist dietitians to practise in socially just ways more broadly, through expanding their awareness of social justice issues. A focus on cultural awareness and cultural safety at the individual level only has been critiqued due to an inability of this approach to address systemic causes of social injustice^(33)^. Structural competence is an alternate approach to cultural competence, which goes beyond an individual focus to actively incorporate structural factors, such as infrastructure, economy and institutional influence, into how health practitioners work with clients^(9,33)^. This approach requires health professionals to understand how health inequities are produced by social and structural forces operating beyond the individual level^(34)^. As such, the structural competence approach could be considered in Australia and Canada, alongside broader recognition of peoples experiencing marginalisation to assist in promoting social justice principles in dietetics by reorienting to broader, structural factors. Intersectionality is another approach that has overlapping principles with structural competence and may assist with reorienting nutrition professionals to a more socially just approach^(35)^. However, there is currently no framework to guide socially just dietetics action, which presents a challenge for dietitians in both Australia and Canada as there is no guidance on how to practise in socially just ways. As such, any adaptations to the incorporation of social justice competencies in dietetics regulatory documents should also be coupled with frameworks or guidelines that support the teaching and assessment of teaching and assessment of dietetics. This will ensure dietitians are provided with practical direction on how to practise in socially just ways, thus contributing to increased social justice action in public health nutrition^(24)^.

### Strengths and limitations

This study is the first to explore how and to what extent social justice is incorporated into dietetic regulatory documents. It provides foundational insight into how social justice is framed in dietetic regulation and therefore provides evidence and rationale for further investigation in this area. Since social justice is highly contextual, the interpretation of social justice terms was based on the researchers’ experience and worldview and may have been interpreted differently by other researchers. This reinforces the importance of developing a standard definition of social justice for the public health nutrition discipline. Trustworthiness of data was managed through researcher reflexivity. This was incorporated into the research project through discussion about codes and themes with the research team. Content analysis was appropriate to achieve the aim and purpose of this study; however, the specific structure of documents and the social contexts in which they are used were not analysed. As such, this study did not address the processes and power relationships that impact the construction of knowledge and policy outcomes^(36)^. Investigating how power structures have influenced the language used and how this impacts the dietetic profession may be of benefit to achieving systemic social justice action for the profession^(36)^. It is acknowledged in the Australian documents that there are some additional documents that contain social justice terms that were not included in the content analysis, for example, the Guide that accompanies the National Competency Standards^(37)^. Furthermore, in line with similar studies which used a content analysis method^(22,38,39)^, we reported raw frequency accounts, with the purpose of getting a sense of how frequently a concept was mentioned in a document. However, this does not take into account that longer documents might have more social justice terms simply by being longer.

## Conclusion

This study is the first to investigate the incorporation of social justice in dietetic regulatory documents in Australia and Canada. The results from this study reveal that in the profession of nutrition and dietetics, social justice is currently framed most commonly in a way that focuses on self-awareness and individualistic care in dietetic regulatory documents. This approach fails to address the systemic nature of social injustice and fails to provide the guidance and governance required to achieve social justice action. Furthermore, the framing of social justice across both countries’ documents was not synonymous with core social justice principles. As such, the dietetics profession needs to re-examine how social justice is framed in regulatory documents, include directives for social justice action and recognise how this may look different in different areas of dietetic practice, including public health nutrition. Developing a clear and consistent definition of what social justice is is a critical first step in achieving this goal. Improving the incorporation of social justice in dietetics regulatory documents and providing explicit guidance on how to practise in socially just ways across all areas of nutrition and dietetics will support dietitians to practise in more socially just ways, thus supporting the advancement of social justice in all areas of nutrition and dietetics including public health nutrition.
